# Identification and validation of the VEGF/p38MAPK/HSP27 pro-tumor inflammatory pathway: screening of active components from *Patrinia villosa* and evaluation of their drug-likeness

**DOI:** 10.3389/fimmu.2025.1631031

**Published:** 2025-08-14

**Authors:** Xiaochen Li, Yang Ju, Xinxin Yang, Tianjiao Li, Shuai Wang, Yongrui Bao, Xiansheng Meng

**Affiliations:** ^1^ College of Pharmacy, Liaoning University of Traditional Chinese Medicine, Dalian, China; ^2^ Liaoning Province Modern Chinese Medicine Research Engineering Laboratory, Dalian, China; ^3^ Liaoning Multi-dimensional Analysis of Traditional Chinese Medicine Technical Innovation Center, Dalian, China; ^4^ Department of Bone Tumor and Orthopaedics, Shenyang Orthopaedic Hospital, Shenyang, China; ^5^ Shenyang Key Laboratory for Causes and Drug Discovery of Chronic Diseases, Shenyang, China

**Keywords:** colorectal cancer (CRC), *Patrinia villosa* Juss. (PV), pro-tumor inflammatory, VEGF/p38MAPK/HSP27, microscale thermophoresis (MST), drug-likeness

## Abstract

**Background:**

Colorectal cancer (CRC) remains a leading cause of cancer-related mortality worldwide, with pro-tumor inflammation playing a critical role in its initiation and progression. Chronic inflammation acts as a major driving force and a distinct mechanism underlying tumorigenesis. Although previous studies have demonstrated the importance of the VEGF/p38MAPK and p38MAPK/HSP27 signaling pathways in CRC-associated inflammation, a comprehensive understanding of the entire pro-tumor inflammatory mechanism remains incomplete.

**Methods:**

This study combined network pharmacology analysis and *in vivo* pharmacodynamic experiments using a p38MAPK pathway inhibitor to systematically identify and validate the VEGF/p38MAPK/HSP27 pro-tumor inflammatory signaling pathway. Western blotting was used to confirm key target proteins. Molecular docking and microscale thermophoresis (MST) experiments were conducted to screen active compounds from *Patrinia villosa* (PV). Molecular dynamics (MD) simulations evaluated the stability and drug-likeness of compound-target interactions.

**Results:**

Key proteins VEGF, p38MAPK, and HSP27 were identified as critical components of the signaling pathway. Three active compounds rutin, nicotiflorin, and 4,5-dicaffeoylquinic acid (4,5-Dicqa) were found to bind these targets with high affinity. MD simulations supported the stability of these interactions and their potential as drug candidates.

**Conclusion:**

This study provides theoretical and experimental evidence for pharmacological targets involved in pro-tumor inflammation in CRC. The findings offer valuable insights for developing novel anti-inflammatory therapeutics targeting the VEGF/p38MAPK/HSP27 signaling pathway.

## Graphical Abstract

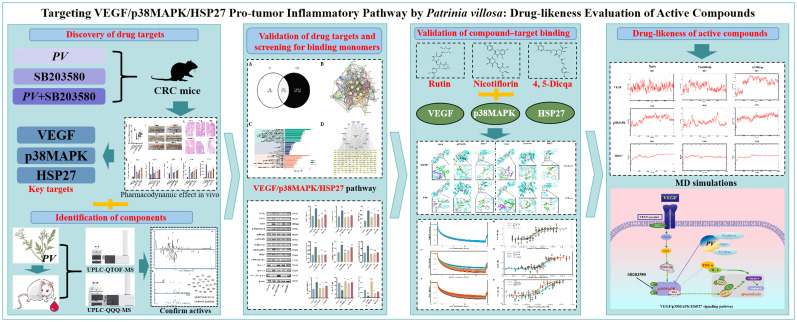

## 1 Introduction

Colorectal cancer (CRC) is the third most common malignancy and the second leading cause of cancer related death worldwide, with over one million new cases diagnosed annually, posing a significant threat to human health (1, 2). Despite recent advances in chemotherapy, targeted therapy, and immunotherapy, the prognosis of CRC remains poor, especially in advanced stage patients, with a five year survival rate still below 65%. Accumulating evidence indicates that chronic inflammation plays a central role in the initiation and progression of CRC (3, 4). Persistent stimulation from reactive oxygen species and pro-inflammatory cytokines within the inflammatory microenvironment can induce DNA damage, aberrant cell proliferation, and angiogenesis, thereby accelerating tumor progression.

In the “inflammation to cancer” pathway, p38 mitogen-activated protein kinase (p38MAPK), a key member of the MAPK family, serves as a critical regulator of inflammatory responses and apoptosis (5–7). Aberrant activation of p38MAPK has been closely linked to the development of multiple cancers (8–10). Studies have confirmed its significantly elevated expression in CRC tissues, and inhibition of this pathway has been shown to effectively suppress tumor progression and enhance chemosensitivity. Previous studies have identified the roles of VEGF/p38MAPK and p38MAPK/HSP27 signaling pathways in CRC related inflammation. However, researchers have not yet fully clarified the complete mechanism. This includes how cells recognize signals at the membrane level, transmit them through the cytoplasm, respond in the nucleus, and regulate apoptosis (9–11). Based on previous research, we hypothesize that p38MAPK plays a central role in CRC. VEGFA acts upstream of p38MAPK and promotes angiogenesis. HSP27 functions downstream, mediating inflammatory responses and regulating apoptosis. Together, these proteins critically regulate tumor progression and represent potential drug targets in CRC.

*Patrinia villosa* (*PV*), a traditional Chinese anti-inflammatory herb, has been documented in historical medical texts and is primarily employed in the treatment of inflammation associated conditions such as intestinal abscesses and enteritis. Modern pharmacological studies have demonstrated that *PV* extract can inhibit the proliferation of CRC cells, induce apoptosis, and modulate inflammation related signaling pathways (12–14). Notably, chronic enteritis is recognized as a significant risk factor for the development of CRC. Repeated inflammatory stimulation of the colonic mucosa can result in epithelial damage, leading to atypical hyperplasia, a precancerous lesion, which may ultimately progress to CRC. This study utilized *PV* as a traditional Chinese medicinal medium to identify and validate the key pharmacodynamic targets-VEGF, p38MAPK, and HSP27 within the VEGF/p38MAPK/HSP27 signaling axis, which are implicated in the regulation of the inflammation tumor process. Furthermore, active compounds with high binding affinity for these targets were screened from the blood entering components of *PV* to elucidate the underlying mechanisms by which they regulate pro-tumor inflammation.

This study integrated network pharmacology and *in vivo* experiments using p38MAPK pathway inhibitors to identify and validate the VEGF/p38MAPK/HSP27 signaling axis involved in pro-tumor inflammation. Western blot analysis confirmed VEGF, p38MAPK, and HSP27 as key regulatory targets. Molecular docking and microscale thermophoresis (MST) revealed that Rutin, Nicotiflorin, and 4,5-Dicqa exhibit strong binding affinities to these targets. Molecular dynamics (MD) simulations further verified the binding stability and drug potential of these compounds. This research lays a theoretical foundation for developing novel therapeutics targeting pro-tumor inflammation.

In summary, this study systematically elucidated the interactions between active components and key pharmacological targets, and identified representative compounds with strong binding affinities to anti pro-tumor inflammatory targets. These findings provide a theoretical foundation and experimental support for anti-inflammatory strategies in tumor intervention and offer new insights into the development of targeted therapeutics from traditional Chinese medicine.

## 2 Materials and methods

### 2.1 Reagents and chemicals

The entire dried *PV* herb was sourced from Guangxi, China, and the medicinal material was authenticated by Professor Jiankui Zhang at Liaoning University. Aladdin supplied Azoxymethane (AOM), and Dextran sodium sulfate (DSS) was obtained from Meilunbio. Chlorogenic acid, caffeic acid, isoorientin, rutin, isovitexin, scutellarin, nicotiflorin, 4,5-Dicqa, ferulic acid, camellia side A, salicylic acid, luteolin, apigenin, gonzalitosin I (5-hydroxy-7, 3’, 4’ -tri methoxy flavone) and ursolic acid (purity > 98%) were purchased from National Institutes for Food and Drug Control (Beijing, China). The test kits, including CA72-4, CA19-9, and CEA were obtained from a biotechnology company in Shanghai, China. IL-6 and TNF-α ELISA Kit were acquired from Solarbio Life Sciences, situated in Beijing, China. Antibodies against β-actin, VEGFA, CDC42, ASK1, p38MAPK, p-p38MAPK, MAPKAPK2, HSP27, Caspase-9, and Caspase-3 were obtained from Proteintech (Wuhan, China). The antibody against p-MAPKAPK2 was purchased from Zenbio (Chengdu, China), the antibody against p-HSP27 (Ser82) was from Cell Signaling Technology (Massachusetts, USA), and the antibody against p-MKK3/MKK6 was from Affinity Biosciences (Jiangsu, China). Human recombinant VEGFA (29714-HNAH), p38MAPK (10081-H07B), HSP27 (10351-H08E) were procured from Sino Biologica in Beijing, China.

### 2.2 *PV* extract

In this study, *PV* was extracted as described in the literature (15). The extract was further purified using a large pore resin column (HPD-300) with 70% ethanol elution. Thereafter, the eluate was collected, vacuum concentrated, and dried to obtain *PV* powder, with a yield of 3.00%.

### 2.3 Establishment of the CRC mouse model and drug administration

A total of 48 SPF grade female C57BL/6 mice (20 ± 2 g) were randomly divided into six groups, with eight mice in each group. Except for the blank group, the mouse models of CRC were established using the previous method (16). After modeling, each group was given the following doses, 5-fluorouracil (5-Fu) (50 mg/kg) (17, 18), *PV* extract (58.5 mg/kg), SB203580 (10 mg/kg) (19), *PV* extract combined with the SB203580 (1.17 mg/20 g + 10 mg/kg), the blank group and the model group were provided with normal saline (0.9%) for 4 consecutive weeks. After 4 week treatment, blood samples were gathered via retro orbital sinus puncture. A part of tissues was taken for pathological examination and Western blot analysis. The detection method is the same as the previous study (16).

### 2.4 Qualitative and quantitative analysis of *PV* extract

The concentration of the *PV* extract in the test solution was 0.5 mg/mL. The *PV* extract analysis was performed using an Agilent 1290 UPLC system and a 6550 QTOF mass spectrometer. Chromatographic separation was achieved with an Agilent poroshell SB-C18 column at 30°C, a flow rate of 0.4 mL/min, and a 0.5 μL injection volume. In positive ion mode, the mobile phase was 0.1% formic acid water (A) and acetonitrile (B), with a gradient elution of 0–40 minutes, 5-100% B. In negative ion mode, the mobile phase A was water, following the same gradient. The mass spectrum parameters were analyzed according to the conventional conditions of the chemical components of traditional Chinese medicine (15). Utilizing UPLC-QQQ-MS analysis, the primary chemical elements in *PV* were quantitatively examined, following the absorption of prototype components in blood. The process of chromatographic separation utilized an Agilent 1290 UPLC system in conjunction with an Agilent 6495 QQQ mass spectrometer. Separation of the sample utilized the Agilent poreshell SB-C18 column (100 mm × 4.6 mm, 2.7 μm). The chromatographic analysis conditions were consistent with UPLC-Q-TOF-MS. Nitrogen gas served dual roles as auxiliary and sheath gases, flowing at a rate of 11 liters per minute. The dry gas temperature was set at 350°C and the nebulizer pressure at 45 psi. Detailed mass spectrometry parameters for quantitative analysis of each component are provided in **Table 1**.

**Table 1:** The identification of the chemical constituents in *PV*.

NO.	Compound	RT (min)	Mass	Product ion (m/z)	Collision energy (eV)
1	Chlorogenic acid	8.397	352.3	190.2	10
2	Caffeic acid	9.769	179.0	135.2	11
3	Isoorientin	10.065	447.6	357.1	20
4	Rutin	11.051	609.0	300.0	36
5	Isovitexin	11.275	431.6	309.9	20
6	Scutellarin	11.705	460.6	285.0	10
7	Nicotiflorin	11.986	593.4	284.6	33
8	4,5-Dicqa	12.290	515.5	353.2	25
9	Ferulic acid	12.530	193.0	178.2	13
10	Camelliaside A	12.904	761.6	618.7	10
11	Salicylic acid	15.294	136.6	93.3	13
12	Luteolin	16.492	284.8	133.0	34
13	Apigenin	18.533	269.4	148.7	34
14	Gonzalitosin I	26.747	328.0	264.3	8
15	Ursolic acid	38.387	454.8	406.4	50

### 2.5 Qualitative and quantitative analysis of *PV* derived constituents in rat plasma

SPF male Sprague Dawley (SD) rats, weighing 200 g ± 20 g were assigned into two groups: blank and treatment, each comprising 8 rats. Rats assigned to the treatment cohort were administered *PV* extract orally at a dosage of 8.1 mg per 200 g for a duration of seven consecutive days, following the guidelines of the 2020 Chinese Pharmacopoeia and adjusting for the conversion of body surface area from humans to rats. Rats in the blank group were administered distilled water. Food was withheld 12 hours before the final administration. Plasma samples were collected and mixed with pre cooled methanol: acetonitrile (volume ratio, 3:1) at a proportion of 1:3. Blood sample treatment was carried out according to the previous conditions of the research group (20). The main chemical components in rat plasma were quantitatively analyzed using UPLC-QQQ-MS analysis. The analysis conditions were the same as the *PV* extract.

### 2.6 Establishment and data processing of molecular networking

First, the collected mass spectra were preprocessed with ProteoWizard. Subsequently, these were separately uploaded to GNPS platform. Then, the visualized molecular network was obtained, and data were processed using Cytoscape 3.7.2 in conjunction with accurate molecular weights. Eventually, the *PV* effective component network was merged with the plasma administration network to establish the MN.

### 2.7 Network pharmacology analysis

The initial step involved inputting the 15 components infused with blood into the UniProt database to acquire UniProt IDs for the anticipated targets. Subsequently, biological targets pertinent to CRC were curated from sources such as GeneCards, OMIM, TTD, and Disgenet. The targets associated with the potential active ingredients for CRC, along with those for *PV*, were then uploaded into the Venn database. The intersection targets related to the MAPK pathway were imported into the STRING database to extract insights regarding protein-protein interaction (PPI) networks. The PPI data was visualized by being processed through Cytoscape 3.7.2, where a PPI network was formulated to identify prospective core targets for further exploration. Ultimately, a component-disease-pathway network was constructed utilizing Cytoscape 3.7.2 software.

### 2.8 Western blot analysis

The extraction of total protein was performed by tissue homogenization and lysis, followed by centrifugation at 4°C and 12000 rpm for 5 min. Separation gel and concentration gel were prepared according to the molecular weight of target protein and allowed to solidify at room temperature. Subsequently, samples were loaded, electrophoresed, transferred to a membrane, and blocked. Membranes were incubated overnight at 4°C with primary antibodies against: β-actin (1:10000),VEGFA (1:500), CDC42 (1:500), ASK1 (1:500), p-MKK3/MKK6 (1:500), p38MAPK (1:2000), p-p38MAPK (1:1000), MAPKAPK2 (1:500), p-MAPKAPK2 (1:500), HSP27 (1:5000), p-HSP27 (Ser 82) (1:1000), Caspase-9 (1:300), Caspase-3 (1:500). After three washes with TBST on a shaker, secondary antibodies were added for incubation. The membranes were then washed again with TBST, and protein signals were visualized using an electrochemiluminescence system (ECL) system. Band intensities were quantified with ImageJ, and statistical analysis was conducted using GraphPad Prism 5.

### 2.9 Docking scores and MST assay

The active constituents present in *PV* and SB203580 were integrated with principal targets through the molecular docking technique, resulting in the acquisition of a binding energy ranking table. The top three active ingredients, *PV* extract and SB203580, were selected for further MST analysis. MST was performed between ligands (*PV* extract, SB203580 and the top three active ingredients for overall affinity score) and human recombinant VEGFA, p38MAPK, HSP27, respectively. Briefly, the target (VEGFA, p38MAPK, HSP27) was labeled with Monolith™ RED-NHS (#23L011_026, NanoTemper Technologies GmbH, Munich, Germany). Subsequently, there were 16 consecutive dilutions of five ligands, and the dilution concentrations are provided in the Supplementary. The labeled human VEGFA, p38MAPK, and HSP27 were mixed with ligands respectively. The samples were introduced within the NanoTemper Monolith™NT (NanoTemper Technologies GmbH, Munich, Germany). Utilizing MO. Affinity Analysis V3.0.5, a curve for fitting was created, and the Kd was subsequently determined (21).

### 2.10 Molecular docking visualization

The 2D structures of nicotiflorin, rutin, 4,5-Dicqa, SB203580 were retrieved from the PubChem database and converted into three dimensional forms using Chem3D software following energy minimization. Additionally, core target crystal structures were obtained from the RCSB Protein Data Bank and prepared through removing water molecules and residues, while supplementing hydrogen atoms with the PyMOL software. The process of molecular docking utilized AutoDockTools 1.5.7 software, and the results were visualized using PyMOL software (22).

### 2.11 MD simulations

To further validate the structural stability and interaction characteristics of the active compound target protein complexes, MD simulations were performed for the docked complexes of the selected active compounds (rutin, nicotiflorin, and 4,5-Dicqa) with the target proteins VEGF, p38MAPK, and HSP27. The simulations were conducted using the GROMACS software package. Ligand parameters were processed under the CHARMM27 force field, and the system was solvated using the TIP3P water model. Energy minimization was carried out using the steepest descent algorithm for 50,000 steps, followed by equilibration phases of 100 ps under constant volume (NVT) and constant pressure (NPT) conditions. Subsequently, 100 ns production simulations were performed on the equilibrated complexes.

### 2.12 Statistical analysis

Data are presented as mean ± standard deviation. Statistical analysis was performed with *p* < 0.05 considered statistically significant, and *p* < 0.01 indicating highly significant differences.

## 3 Results

### 3.1 Physiological parameters in mice

**Figure 1:**
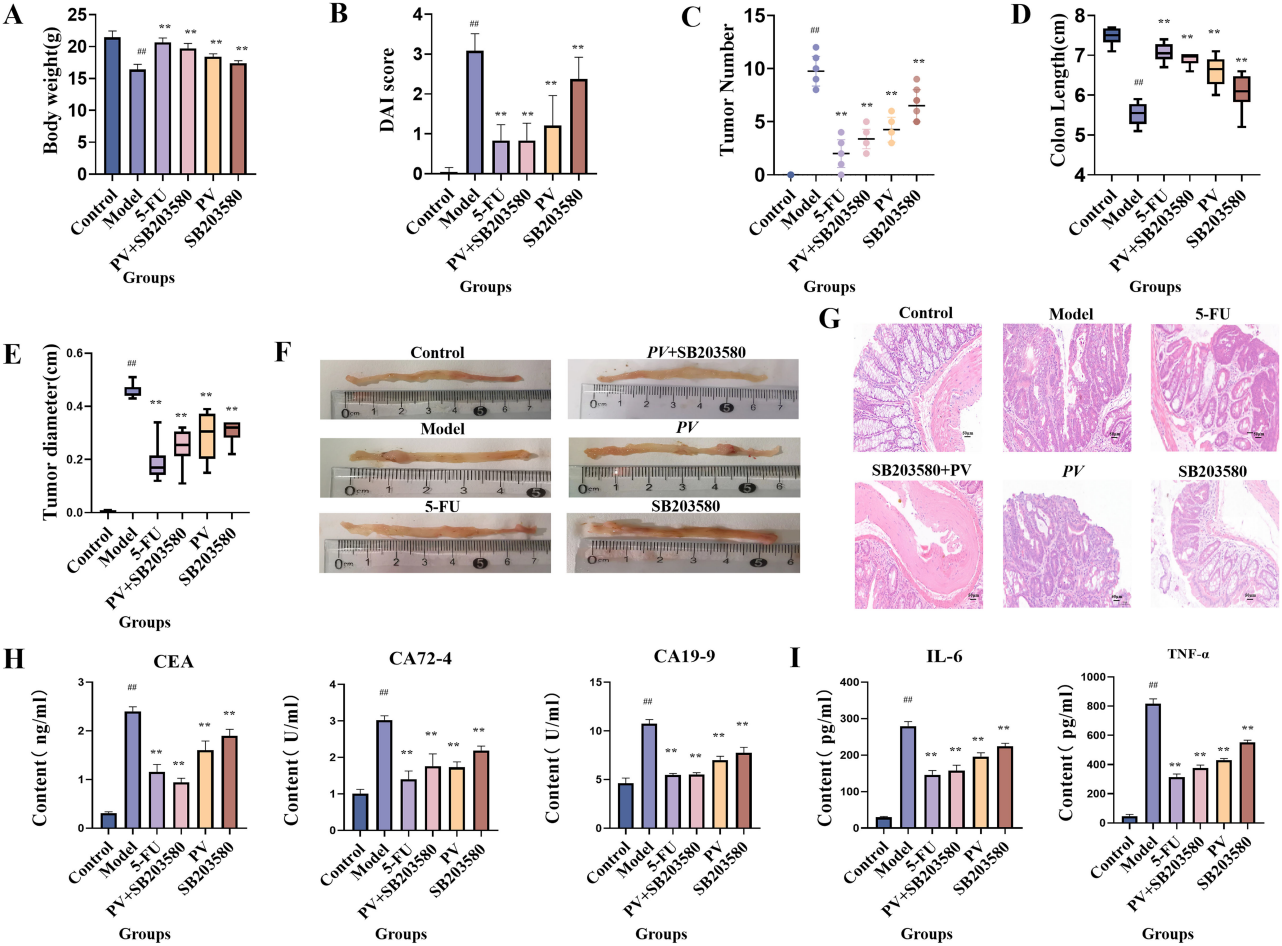
Pharmacological effect test. **(A)** Body weight of mice. **(B)** DAI of mice. **(C)** Macroscopic tumors number. **(D)** Colon length. **(E)** Tumor diameter. **(F)** Colonic appearance. **(G)** Histopathological sections (20×). **(H)** Biochemical indexes. **(I)** Inflammatory index. ^##^comparing with blank group, *p* < 0.01. **comparing with model group, *p* < 0.01.

Within the six mouse groups, there was a notable reduction in the body weight of mice in the model group after four weeks of treatment compared to the control group (*p* < 0.01), whereas in other treatment groups, there was a significant increase in body weight relative to the model group (*p* < 0.01) in **Figure 1A**. In the experiment, body weight, fecal characteristics, and presence of blood in the feces were found to calculate the disease activity index (DAI) (23). Differences between treatment group and model group were statistically significant (*p* < 0.01), as shown in **Figure 1B**.

### 3.2 Observation of histopathological tissues in mice

As depicted in **Figure 1C**, there was a notable decrease in tumor count across all treatment groups (*p* < 0.01). In the model group, mice exhibited a reduced colon length compared to the blank group (*p* < 0.01), whereas each treatment group showed a notable recovery in **Figure 1D**. The tumor diameter of mice in each treatment group also dramatically decreased (*p* < 0.01) in **Figure 1E**. Moreover, the blank group did not show any sign of tissue carcinoma, while the model group exhibited substantial tumor formation in **Figure 1F**. By employing hematoxylin and eosin staining techniques, the control mouse group exhibited a normal, inflammation free colon devoid of any noticeable irregularities. Conversely, the model group revealed characteristics of poorly differentiated adenocarcinoma. Significantly, the model group exhibited crypt hypoplasia and adenoma hyperplasia, characterized by a higher nucleus/cytoplasmic ratio. Additionally, the model group showed increased fibrous interstitial zones and necrotic regions, along with a notable presence of inflammatory cells in the tumor. However, following intervention with various treatments, the above pathological changes were significantly improved in **Figure 1G**. Furthermore, the combined use of *PV* extract with the p38MAPK pathway inhibitor in mice yielded superior outcomes to the administration of *PV* extract or the inhibitor alone. SB203580, as a p38MAPK pathway inhibitor, can suppress the activation of p38MAPK and thereby block the downstream effects of p38MAPK signal transduction. The combined use of *PV* extract and the p38MAPK pathway inhibitor further enhances the inhibitory effect on the p38MAPK pathway, which can thus more effectively suppress CRC cell growth and dissemination, indicating that the pharmacological efficacy of *PV* is related to the p38MAPK pathway.

### 3.3 Biochemical parameters in mice

ELISA was used to measure the concentrations of CEA, CA19-9, and CA72-4, as well as IL-6 and TNF-α in mouse serum. Compared to the control group, the model group exhibited a significant increase in CEA, CA19-9, and CA72–4 levels (*p* < 0.01). All treatment groups showed a notable reduction in these tumor markers compared to the model group (*p* < 0.01). Moreover, the combined administration of *PV* extract with the p38MAPK pathway inhibitor led to a more pronounced decrease in CEA, CA19-9, and CA72–4 levels compared to treatment with *PV* or the inhibitor alone (**Figure 1H**). Similarly, the model group showed a significant elevation in the levels of IL-6 and TNF-α compared to the control group (*p* < 0.01), indicating an inflammatory response. All treatment groups significantly reduced IL-6 and TNF-α levels (*p* < 0.01), with the combination of *PV* extract and the p38MAPK pathway inhibitor demonstrating the most substantial reduction (**Figure 1I**).

### 3.4 Chemical constituents from *PV* extract absorbed into rat plasma

**Figure 2:**
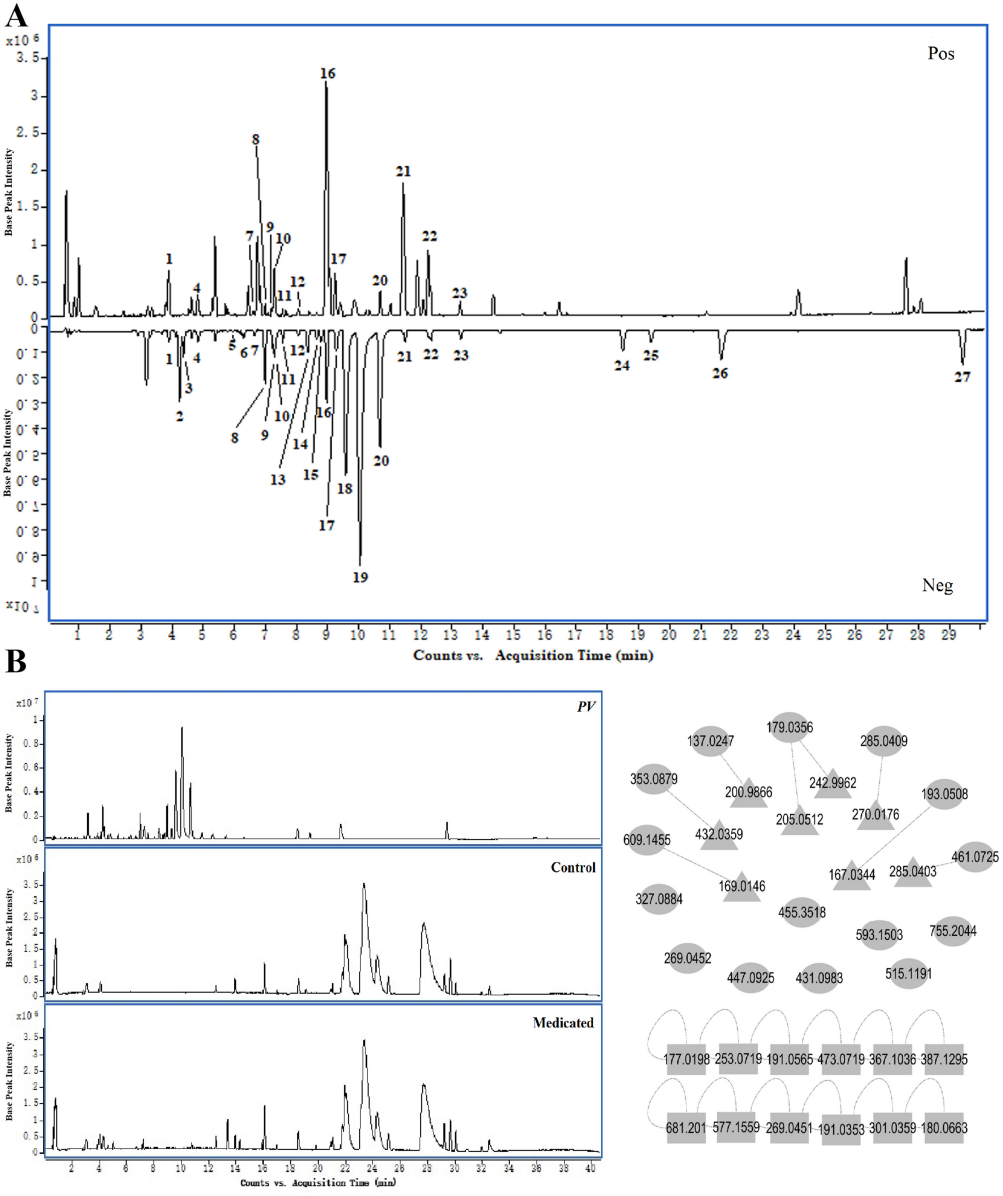
Chemical components. **(A)** BPC of *PV* extract in positive and negative ion modes. **(B)** MN of active components in *PV* extract. (○, prototypal compounds; △, related metabolites; □, Not entered blood).

The Base Peak Chromatogram (BPC) of *PV* extract is illustrated in **Figure 2A**, highlighting the active constituents concentrated within a retention time of 0–30 minutes. Consequently, the BPC pertaining to this specific timeframe is presented exclusively. By constructing a MN of the chemical constituents present in *PV* extract and the absorbed bloodstream components after oral administration in rats, its pharmacological components were analyzed. A total of 27 active compounds were identified. Among them, 15 were detected in the bloodstream of rats, including chlorogenic acid, caffeic acid, isoorientin, rutin, isovitexin, scutellarin, nicotiflorin, 4,5-Dicqa, ferulic acid, camelliaside A, salicylic acid, luteolin, apigenin, gonzalitosin I, and ursolic acid. In addition, eight relevant metabolites were identified, as shown in **Table 2** and **Figure 2B**.

**Table 2:** Chemical components.

No.	RT (min)	Formula	Ion type	Theoretical mass (Da)	Calculated mass (Da)	Mass error (ppm)	Identified compounds	Medicinal materials	Enter the blood
P1^*^	3.894	C_16_H_18_O_9_	[M-H]^-^	353.0878	353.0879	0.28	Chlorogenic acid	✓	✓
P2	4.241	C_9_H_6_O_4_	[M-H]^-^	177.0193	177.0198	2.82	Daphentin	✓	–
P3^*^	4.374	C_9_H_8_O_4_	[M-H]^-^	179.0350	179.0356	3.34	Caffeic acid	✓	✓
P4	4.837	C_11_H_12_O_4_	[M+COOH]^-^	253.0718	253.0719	0.40	Caffeic acid ethyl ester	✓	–
P5^*^	5.979	C_21_H_20_O_11_	[M-H]^-^	447.0933	447.0925	-1.79	Isoorientin	✓	✓
P6	6.310	C_7_H_12_O_6_	[M-H]^-^	191.0561	191.0565	2.09	Quinic acid	✓	–
P7	6.657	C_22_H_18_O_12_	[M-H]^-^	473.0725	473.0719	-1.27	Cichoric acid	✓	–
P8^*^	6.988	C_27_H_30_O_16_	[M-H]^-^	609.1461	609.1455	-0.98	Rutin	✓	✓
P9^*^	7.220	C_21_H_20_O_10_	[M-H]^-^	431.0984	431.0983	-0.23	Isovitexin	✓	✓
P10^*^	7.302	C_21_H_18_O_12_	[M-H]^-^	461.0725	461.0726	0.22	scutellarin	✓	✓
P11^*^	7.550	C_27_H_30_O_15_	[M-H]^-^	593.1512	593.1503	-1.52	Nicotiflorin	✓	✓
P12^*^	8.063	C_25_H_24_O_12_	[M-H]^-^	515.1195	515.1191	-0.78	4,5-Dicqa	✓	✓
P13^*^	8.361	C_10_H_10_O_4_	[M-H]^-^	193.0506	193.0508	1.04	Ferulic acid	✓	✓
P14	8.659	C_17_H_20_O_9_	[M-H]^-^	367.1035	367.1036	0.27	5-O-Feruloylquinic acid	✓	–
P15	8.791	C_17_H_24_O_10_	[M-H]^-^	387.1297	387.1295	-0.52	Geniposide	✓	–
P16^*^	8.973	C_33_H_40_O_20_	[M-H]^-^	755.2040	755.2044	0.53	Camelliaside A	✓	✓
P17^*^	9.288	C_7_H_6_O_3_	[M-H]^-^	137.0244	137.0247	2.19	Salicylic acid	✓	✓
P18	9.602	C_29_H_34_O_15_	[M+CH_3_COO]^-^	681.2036	681.2010	-3.82	Pectolinarin	✓	–
P19	10.049	C_27_ H_30_ O_14_	[M-H]^-^	577.1563	577.1559	-0.69	Chrysin 7-O-β-gentiobioside	✓	–
P20^*^	10.694	C_15_H_10_O_6_	[M-H]^-^	285.0405	285.0409	1.40	Luteolin	✓	✓
P21	11.505	C_15_H_10_O_5_	[M-H]^-^	269.0455	269.0451	-1.49	Aloeemodin	✓	–
P22^*^	12.316	C_15_H_10_O_5_	[M-H]^-^	269.0455	269.0452	-1.12	Apigenin	✓	✓
P23	13.292	C_10_H_8_O_4_	[M-H]^-^	191.0350	191.0353	1.57	Scopoletin	✓	–
P24^*^	18.504	C_18_H_16_O_6_	[M-H]^-^	327.0874	373.0917	3.06	Gonzalitosin I	✓	✓
P25	19.414	C_15_H_10_O_7_	[M-H]^-^	301.0354	301.0359	1.66	Quercetin	✓	–
P26	21.664	C_9_H_11_NO_3_	[M-H]^-^	180.0666	180.0663	-1.67	5-(1′-hydroxyethyl)- methyl nicotinate	✓	–
P27^*^	29.440	C_30_H_48_O_3_	[M-H]^-^	455.3531	455.3518	-2.85	Ursolic acid	✓	✓
M1	3.115	C_16_H_17_O_12_S	[M-H]^-^	432.0368	432.0359	-2.08	Chlorogenic acid sulfate	–	✓
M3	4.108	C_9_H_8_O_6_S	[M-H]^-^	242.9969	242.9962	-2.88	Caffeic acid sulfate	–	✓
M3	5.051	C_11_H_10_O_4_	[M-H]^-^	205.0506	205.0512	2.93	Caffeic acid derivative	–	✓
M8	2.453	C_7_H_6_O_5_	[M-H]^-^	169.0142	169.0146	2.37	Rutin-derived gallic acid	–	✓
M13	4.571	C_8_H_8_O_4_	[M-H]^-^	167.0350	167.0344	-3.59	Ferulic acid-derived vanillic acid	–	✓
M17	8.129	C_7_H_6_O_5_S	[M-H]^-^	200.9863	200.9866	1.49	Salicylic acid sulfate	–	✓
M20	10.701	C_14_H_7_O_6_	[M-H]^-^	270.0170	270.0176	2.22	Luteolin-derived oxidation product	–	✓

*Verified by comparison with reference standards; P, prototypal compounds; M, related metabolites. √, the substance was detected; –, the substance was not detected.

### 3.5 Analysis of 15 prototype chemical constituents in *PV* extract and rat plasma

The content of chlorogenic acid, caffeic acid, isoorientin, rutin, isovitexin, scutellarin, nicotiflorin, 4,5-Dicqa, ferulic acid, camellia side A, salicylic acid, luteolin, apigenin, gonzalitosin I and ursolic acid in *PV* extract and after absorption in the blood of rats was determined in MRM mode. Calculations of each compound’s composition were derived from their corresponding calibration curves, with the findings displayed in **Table 3**.

**Table 3:** Standard curve and linear range of each compound.

NO.	Compound	*PV* extract				Rat plasma			
		Regression equation	R^2^	Linear range (ng/ml)	Mean ± SD (ng/ml)	Regression equation	R^2^	Linear range (ng/ml)	Mean ± SD (ng/ml)
1	Chlorogenic acid	y = 123.92x + 433.79	0.9951	14.68-43000	3446.64 ± 52.19	y = 0.0218x + 0.2705	0.9986	0.55-2080	62.61 ± 1.92
2	Caffeic acid	y = 4.8379x + 9.0451	0.9951	10.58-31000	2364.65 ± 14.51	y = 0.0016x - 0.0023	0.9991	0.31-1170	357.66 ± 29.22
3	Isoorientin	y = 209.99x + 13827	0.9968	27.99-82000	55.97 ± 3.15	y = 0.0719x - 0.0597	0.9968	0.32-490	2.52 ± 0.31
4	Rutin	y = 64.517x + 1997.1	0.9973	21.50-63000	414.65 ± 9.17	y = 0.0675x + 0.0423	0.9962	0.38-36	0.64 ± 0.11
5	Isovitexin	y = 39.429x + 1592.2	0.9994	3.07-9000	10.90 ± 0.96	y = 0.0134x + 0.0458	0.9984	0.18-700	5.55 ± 0.74
6	Scutellarin	y = 42.327x + 843.34	0.9959	9.90-29000	4358.23 ± 37.90	y = 0.0065x + 0.082	0.9947	0.38-1440	257.85 ± 15.86
7	Nicotiflorin	y = 527.8x + 1634.6	0.9996	3.07-9000	120.06 ± 1.40	y = 0.141x + 0.0655	0.9961	0.20-19	0.23 ± 0.04
8	4,5-Dicqa	y = 72.81x + 1535.5	0.9895	7.85-23000	357.21 ± 11.16	y = 0.0222x - 0.01	0.9987	0.66-400	4.08 ± 0.82
9	Ferulic acid	y = 1.2416x + 18.573	0.9946	11.26-33000	99.41 ± 10.20	y = 0.0001x + 0.0059	0.9953	2.53-1540	36.38 ± 6.48
10	Camelliaside A	y = 0.7414x + 31.854	0.9980	7.17-21000	101.36 ± 16.90	y = 0.0005x + 0.0047	0.9968	0.45-277	43.28 ± 5.83
11	Salicylic acid	y = 66.352x + 305.56	0.9998	15.36-45000	114.08 ± 0.84	y = 0.0009x + 0.0325	0.9968	0.44-1680	37.74 ± 3.55
12	Luteolin	y = 179.78x - 559.81	0.9982	4.78-14000	2297.25 ± 60.90	y = 0.0566x + 0.512	0.9982	0.22-850	124.76 ± 10.93
13	Apigenin	y = 225.12x + 1082.7	0.9961	7.85-23000	63.99 ± 3.05	y = 0.0131x - 0.0069	0.9990	0.19-280	13.15 ± 1.21
14	Gonzalitosin I	y = 4.1346x + 1708.6	0.9988	6.14-18000	332.66 ± 43.91	y = 0.0007x + 0.029	0.9953	0.31-960	19.15 ± 4.81
15	Ursolic acid	y = 22.284x - 271.79	0.9929	9.90-29000	16.15 ± 0.23	y = 0.0131x - 0.0069	0.9990	0.34-1300	2.67 ± 0.98

### 3.6 *PV* treatment of CRC through the MAPK signaling pathway

**Figure 3:**
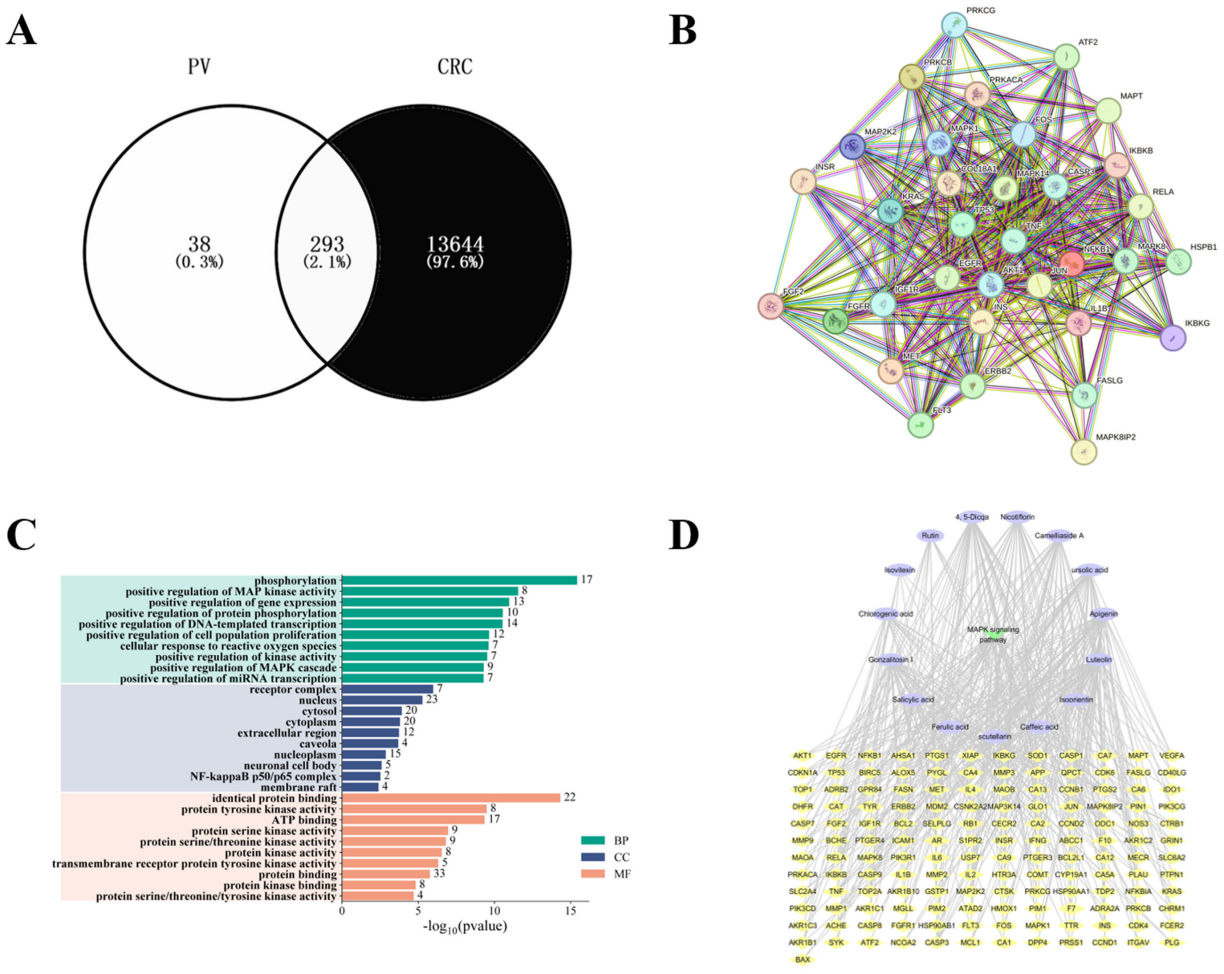
Results of network pharmacology. **(A)** Intersection of *PV* and CRC targets. **(B)** Protein-protein interaction network. **(C)** GO enrichment analysis. **(D)** Compound-target-pathway network construction.

The 15 absorbed components can regulate 293 targets. Additionally, a total of 13,937 CRC related targets were identified from databases such as GeneCards, identifying a total of 293 potential targets for *PV* in CRC treatment were selected (**Figure 3A**). A total of 34 key targets related to the MAPK pathway were selected, and a PPI network was established. Core targets include CASP3, MAPK14 (p38MAPK), VEGFA, and HSPB1 (HSP27), as shown in **Figure 3B**. **Figure 3C** illustrates Gene Ontology (GO) enrichment analysis pertinent to the MAPK signaling pathway, while **Figure 3D** presents a summary of the interactions among components, targets, and pathways. Under the Biological Process (BP) classification, the enrichment analysis of the common target genes predominantly highlights processes such as phosphorylation, the positive modulation of MAP kinase activity, and the enhancement of gene expression. In the Cellular Component (CC) category, significant enrichment was identified in receptor complexes, as well as in the nucleus and cytoplasm. Furthermore, within the Molecular Function (MF) classification, notable enrichment was detected in activities related to protein binding, protein tyrosine kinase activity, and ATP binding.

### 3.7 Western blot analysis of mouse samples

**Figure 4:**
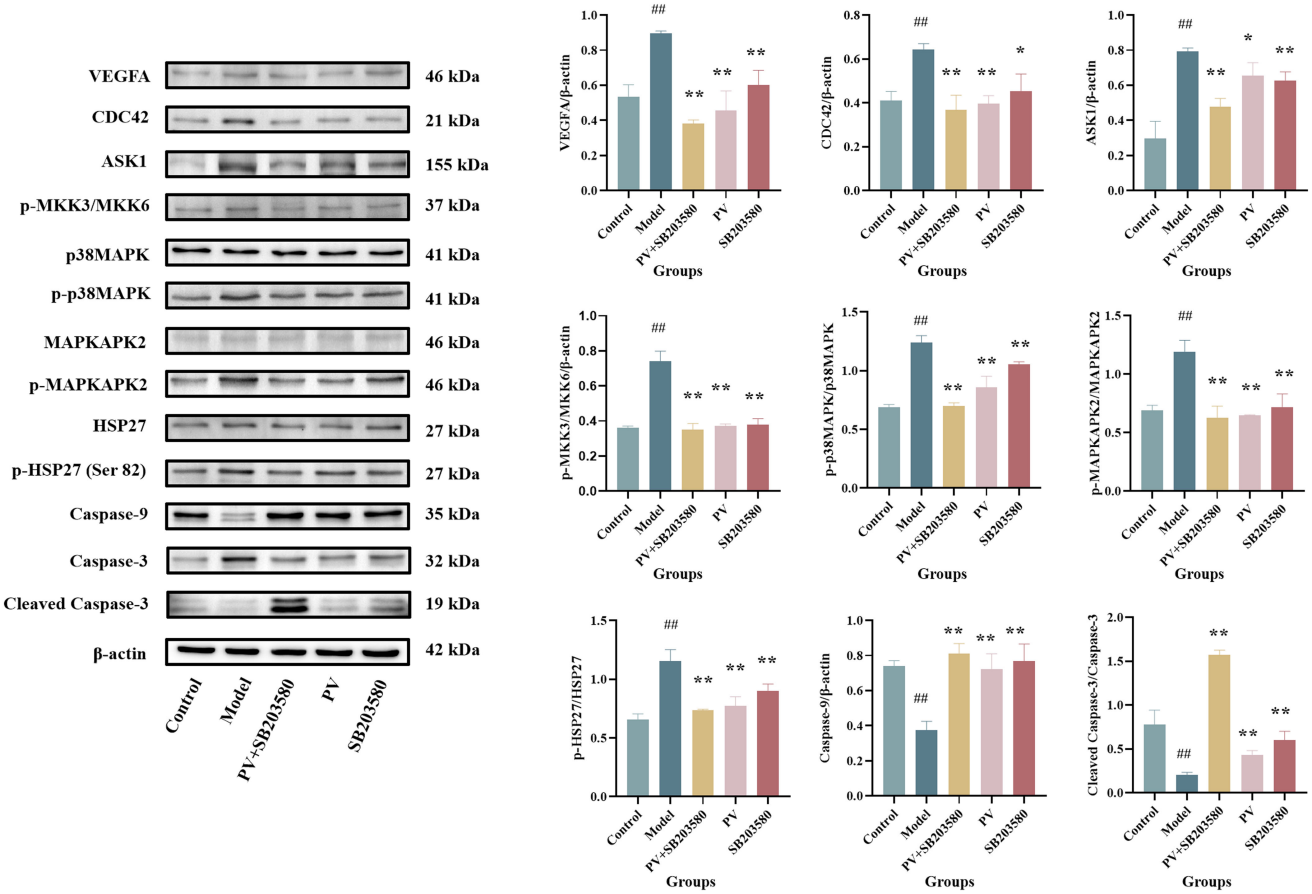
Protein levels in the colon of mice. Values are presented as the mean ± standard deviation (n=3); ^##^compared to the blank group, *p* < 0.01. **compared to the model group, *p* < 0.01.* compared to the model group, *p* < 0.05.

As observed from **Figure 4**, the combined treatment of *PV* extract with p38MAPK inhibitor resulted in a greater reduction in the relative expression levels of VEGFA, CDC42, ASK1, p-MKK3/MKK6, p-p38MAPK/p38MAPK, p-MAPKAPK2/MAPKAPK2, p-HSP27 (Ser82)/HSP27, as well as an increase in Caspase-9 and Cleaved Caspase-3/Caspase-3 compared with *PV* extract or p38MAPK inhibitor alone group. *PV* extract may cooperate with the p38MAPK inhibitor for binding to the same receptors, thereby affecting the expression of upstream and downstream target proteins in the p38MAPK pathway and enhancing the pharmacological activity. This further elucidates the mechanism by which *PV* exerts its pharmacological effects via the regulation of the p38MAPK pathway.

### 3.8 Docking scores analysis and MST analysis

**Figure 5:**
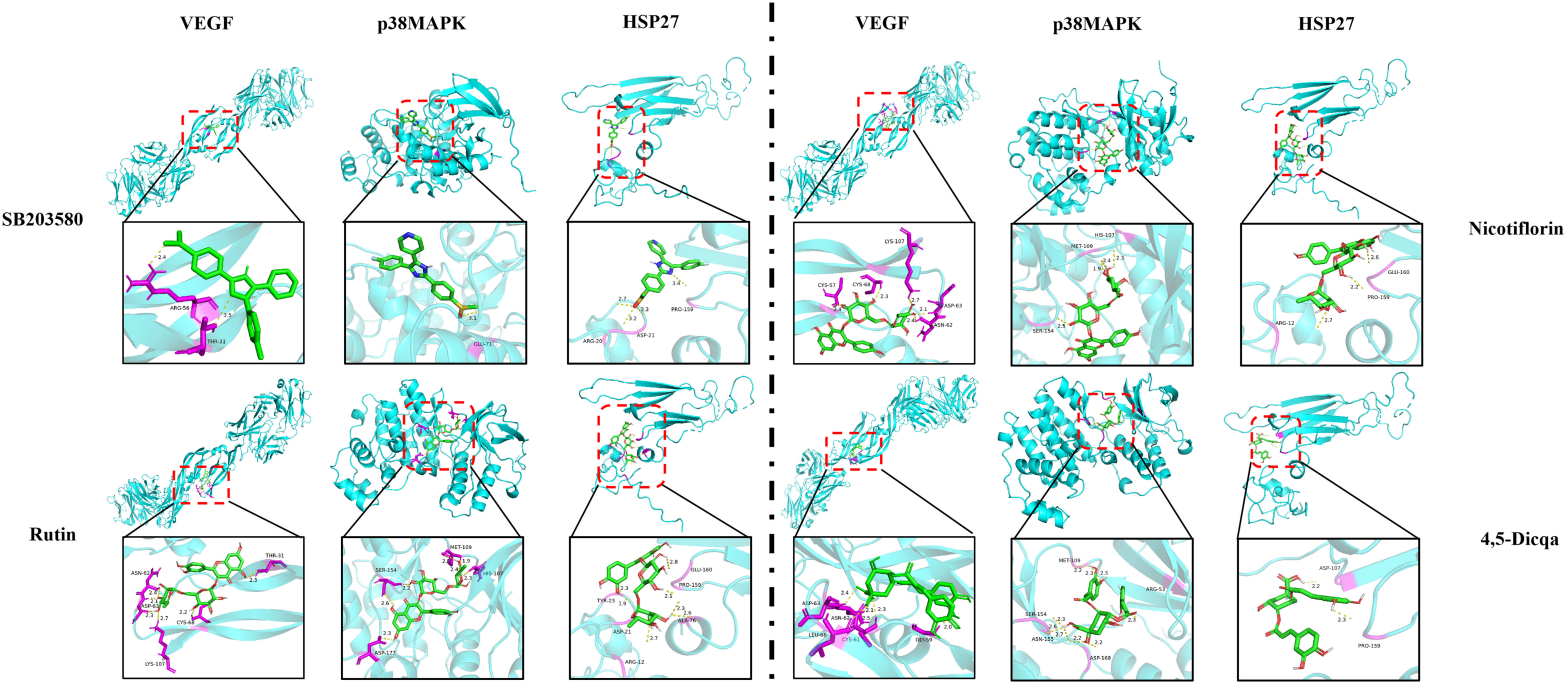
Molecular docking of key targets with bioactive compounds.

**Figure 6:**
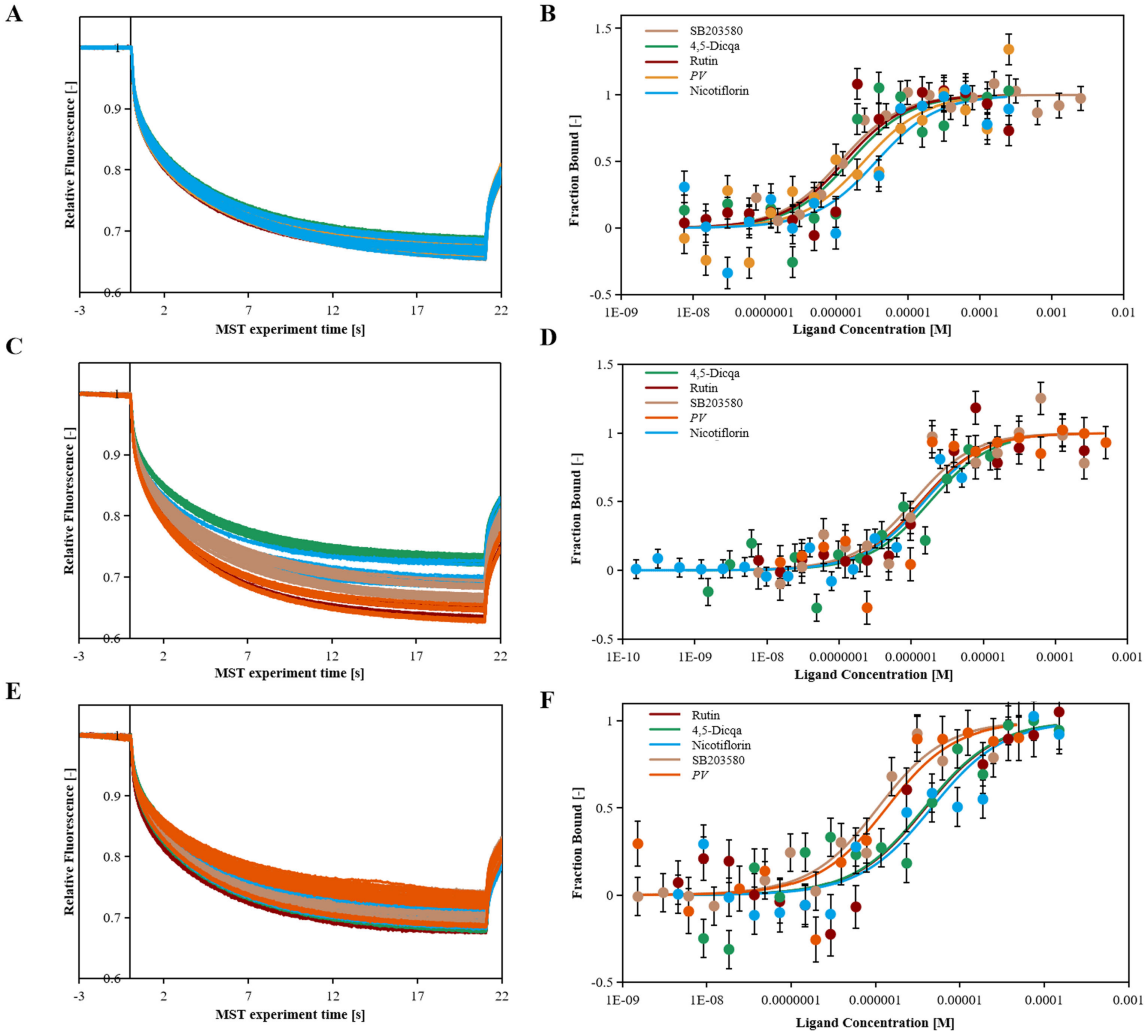
The binding kinetics of ligands and targets. **(A)** Relative fleorescence time curve of the MST process. **(B)** Dissociation curve of SB203580, *PV*, Nicotiflorin, Rutin, 4,5-Dicqa and VEGF protein samples. **(C)** Relative fleorescence time curve of the MST process. **(D)** Dissociation curve of SB203580, *PV*, Nicotiflorin, Rutin, 4,5-Dicqa and p38MAPK protein samples. **(E)** Relative fleorescence time curve of the MST process. **(F)** Dissociation curve of SB203580, *PV*, Nicotiflorin, Rutin, 4,5-Dicqa and HSP27 protein samples.

Using molecular docking technology, the interactions between the 15 blood absorbed components mentioned above and the key targets of the VEGF/p38MAPK/HSP27 signaling pathway were validated. The docking results are shown in **Table 4**. The docking scores of the *PV* active compounds and SB203580 with core target proteins were all less than -5 kcal/mol, indicating a strong binding affinity of *PV* active compounds and SB203580 with core proteins in the p38MAPK pathway. Among them, nicotiflorin, rutin, and 4,5-Dicqa exhibited the highest binding abilities, and the primary findings of molecular docking visualization are presented in **Figure 5**. The results indicate that SB203580 binds to the p38MAPK target at THR-106 and LEU-104. SB203580 is an inhibitor of the p38MAPK pathway, strongly attracting and interacting with the p38 target, thus disrupting the interaction between p38MAPK and its substrates. The inhibition of p38MAPK activity affects both upstream and downstream signal cascades. Notably, 4,5-Dicqa also binds to the p38MAPK target at ARG-173. This finding suggests a common binding site for both SB203580 and 4,5-Dicqa on the p38MAPK protein, which may imply a similar mode of action or interference with the same functional region of p38MAPK. At the same time, rutin binds to the HSP27 target at THR-31, a site also shared by SB203580 and the VEGF target. nicotiflorin and rutin also share the binding site CYS-137 on the HSP27 target with SB203580, indicating that these compounds may exert their effects by modulating VEGF and HSP27 through a common mechanism. Subsequently, MST analysis was conducted to further assess the interaction between ligands (*PV* extract, nicotiflorin, rutin, 4,5-Dicqa, and SB203580) and *in vitro* targets. The dissociation constant (Kd) values are shown in **Figures 6A–F** and **Table 5**. The results demonstrate that the ligands bind to their targets at low micromolar concentrations, validating the experimental predictions of molecular docking and indicating good binding between ligands and receptors.

**Table 4:** Molecular docking results of active ingredients and key targets.

Compound	Estimated ΔG (kcal/mol)		
	VEGF	p38MAPK	HSP27
SB203580	-7.4	-9.9	-10.0
Nicotiflorin	-7.6	-8.6	-10.1
Rutin	-7.5	-8.5	-10.2
4,5-Dicqa	-7.8	-8.6	-9.6
Ursolic acid	-7.5	-7.8	-9.7
Camelliaside A	-7.5	-9.0	-8.4
Scutellarin	-7.7	-8.0	-8.8
Luteolin	-7.2	-8.2	-9.1
Isovitexin	-7.4	-7.5	-9.1
Isoorientin	-7.8	-7.9	-8.1
Chlorogenic acid	-7.0	-7.3	-9.2
Gonzalitosin I	-6.8	-7.5	-9.1
Apigenin	-6.7	-7.8	-8.8
Caffeic acid	-5.6	-6.3	-9.6
Ferulic acid	-5.2	-6.0	-7.0
Salicylic acid	-5.1	-5.8	-6.1

**Table 5:** MST results of ligands and targets.

Compound	Kd (μM)		
	VEGF	p38MAPK	HSP27
SB203580	1.034	0.967	0.915
*PV*	2.315	1.309	1.200
Nicotiflorin	3.476	1.403	4.466
Rutin	1.197	1.270	3.758
4,5-Dicqa	1.430	1.709	3.638

### 3.9 MD simulation of active compounds and target proteins

**Figure 7:**
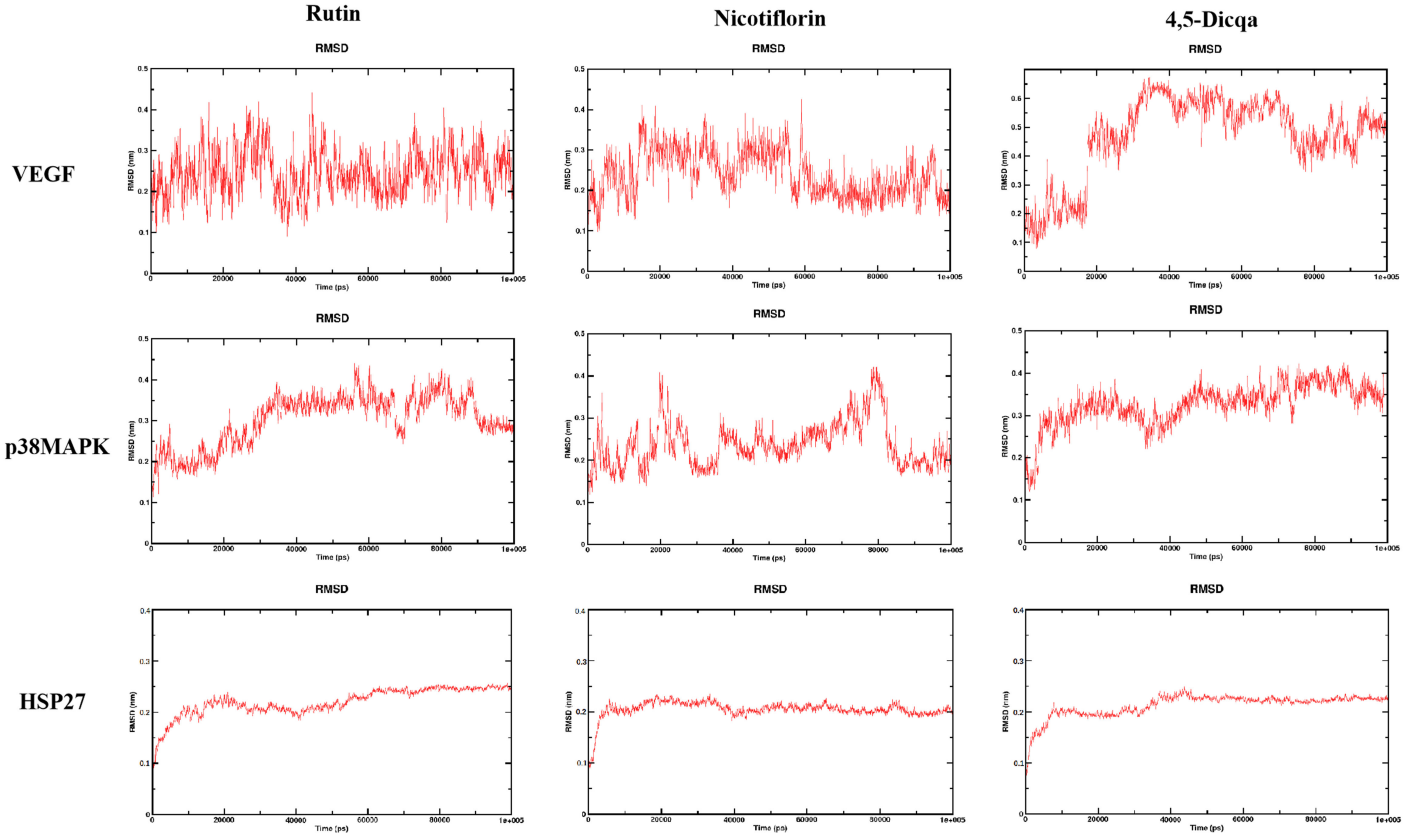
RMSD trajectories of the complexes formed by the three active compounds (Rutin, Nicotiflorin, and 4,5-Dicqa) with VEGF, p38MAPK, and HSP27 over 100 ns MD simulations.

The stability of the protein ligand complexes during the simulation was evaluated using root mean square deviation (RMSD) analysis and corresponding visualization curves. The results indicated that all systems gradually reached stability during the simulation, with no significant conformational drift observed (**Figure 7**).

## 4 Discussion

CRC is one of the most common malignancies of the digestive system, with an increasing trend in both incidence and mortality rates worldwide (24–28). According to epidemiological data, CRC ranks third in incidence and second in mortality among all cancers globally (29, 30). In China, the incidence rate is steadily increasing and affecting younger populations, with approximately 560,000 new cases and 290,000 deaths annually. Tumor progression has long been associated with chronic inflammatory stimulation (31). Persistent inflammation of the intestinal mucosa is a major contributing factor to colorectal carcinogenesis. Inflammatory microenvironments surrounding tumors contain various molecules such as cytokines, chemokines, and prostaglandins, which may cause DNA damage, suppress tumor suppressor genes, and promote proto oncogene overexpression, leading to malignant transformation of cells (32). During chronic inflammation, immune cells such as lymphocytes and macrophages secrete inflammatory mediators including cytokines and chemokines, collectively establishing a pro-tumorigenic inflammatory microenvironment. This tumor microenvironment, shaped by persistent inflammation, contributes to angiogenesis, tumor metastasis, and reduced sensitivity to therapeutic agents. Tumor cells often overexpress pro-inflammatory mediators that help maintain this microenvironment, further promoting inflammation and forming a vicious cycle (33).

The p38MAPK signaling pathway, a crucial member of the MAPK family, can be activated by various inflammatory cytokines and stress signals via dual phosphorylation on tyrosine and threonine residues (34, 35). It plays a pivotal role in regulating inflammation, apoptosis, and other physiological and pathological processes. In particular, the p38MAPK pathway serves as a critical link between inflammation and tumorigenesis. Persistent activation of p38MAPK has been shown to enhance angiogenesis and inhibit apoptosis (36). Our findings reinforce this evidence, demonstrating that *PV* exhibits significant anti-inflammatory and anti-tumor activity in CRC models, likely through modulation of the p38MAPK cascade. The inflammatory microenvironment plays a key role in the initiation, progression, and metastasis of CRC. Recent pharmacological studies have confirmed the notable anti-inflammatory and anticancer effects of *PV*. Based on previous findings that its anti-inflammatory and anti-tumor effects are mediated through pathways including PI3K and p38MAPK, the current study focuses on the p38MAPK pathway (16). By employing an integrative approach that combines network pharmacology with *in vivo* pharmacological studies targeting p38MAPK inhibition, we have identified and confirmed the pivotal regulatory function of the VEGF/p38MAPK/HSP27 signaling axis in CRC associated pro-tumor inflammation. While previous studies have suggested the involvement of both VEGF/p38MAPK and p38MAPK/HSP27 pathways in inflammation related to colorectal cancer, a comprehensive elucidation of the process by which pro-tumor inflammation transitions from membrane signal detection to cytoplasmic transduction and nuclear transcriptional response is still lacking.

*PV*, a traditional Chinese medicine, has long been recorded for its functions of “promoting blood circulation, resolving stasis, and detoxifying swelling” in classical texts such as the *Chinese Pharmacopoeia* (1977 edition), *Compendium of Materia Medica*, and *Changsha Medicine*. Clinically, it is commonly used to treat digestive system disorders including cholecystitis, enteritis, and ulcerative colitis. On the basis of identifying druggable targets with anti-inflammatory and anti-tumor potential, screening for high affinity active monomers from traditional anti-inflammatory medicines and evaluating their drug-likeness may provide a foundation for the discovery of anti-tumor drugs that target inflammation.

Molecular docking analysis revealed that the active compounds within *PV* demonstrated robust binding affinities to critical targets such as VEGF, p38MAPK, and HSP27, primarily through hydrogen bonding, hydrophobic interactions, and electrostatic forces. Specifically, SB203580, a classic p38MAPK inhibitor, formed a stable hydrogen bond with residue GLU-71 within the canonical binding pocket; interactions with VEGF involved hydrogen bond networks and electrostatic attraction with ARG-56 and THR-31. For HSP27, SB203580 formed multiple interactions: hydrophobic and van der Waals forces with PRO-159, hydrogen bonding and electrostatics with ARG-21, and salt bridge formation with ASP-21. Rutin, rich in hydroxyl groups, exhibited strong hydrogen bonding capabilities forming multiple hydrogen bonds with SER-154, ASP-177, HIS-107, and MET-109 in p38 MAPK; hydrogen bonds and hydrophobic interactions with ASN-62, ASP-63, LYS-107, CYS-68, and THR-31 in VEGF; and a network of hydrogen bonds (GLU-160, ARG-21, ASP-21), π-π stacking (TYR-23), and hydrophobic interactions (ALA-76, PRO-159) in HSP27. Nicotiflorin, a small alkaloid with good membrane permeability, formed hydrogen bonds with HIS-107 and SER-154 and hydrophobic interactions at MET-109 in p38MAPK; it interacted with VEGF via hydrogen bonds and hydrophobic forces involving CYS-57, CYS-68, LYS-107, ASP-63, and ASN-62; and formed hydrogen bonds and electrostatic interactions with GLU-160 and ARG-12 in HSP27, with additional van der Waals interactions at PRO-159. 4,5-Dicqa, containing multiple phenolic hydroxyl and carboxyl groups, formed extensive hydrogen bond networks with p38MAPK residues MET-109, SER-154, ASP-168, ASN-155, and ARG-53, complemented by van der Waals forces. It formed stable hydrogen bonds and hydrophobic contacts with ASP-63, LEU-66, CYS-61, ASN-62, and GLY-59 in VEGF; and with PRO-159 and ASP-107 in HSP27. To further evaluate the stability of these ligand-protein complexes, 100-ns MD simulations were conducted for rutin, nicotiflorin, and 4,5-Dicqa with each of the key targets (VEGF, p38 MAPK, and HSP27). RMSD analysis revealed that all ligand target systems maintained high structural stability over the entire 100 ns simulation, with limited fluctuations and no significant conformational shifts, suggesting stable molecular level binding. These results are consistent with the high affinity observed in docking and MST experiments, supporting the potential of these compounds as promising small molecule inhibitors with drug-like properties, laying a solid foundation for future mechanistic studies and drug development based on anti-inflammatory and anticancer traditional medicines.

Despite the encouraging results, this study has several limitations. First, the DSS induced colitis associated CRC mouse model, although widely used, does not fully reflect the complexity and heterogeneity of human CRC. Second, our data highlight the critical role of the VEGF/p38MAPK/HSP27 axis in driving the anti-inflammatory and pro-apoptotic effects, but deeper mechanistic studies are still needed to confirm causality. Finally, future clinical trials are necessary to evaluate the efficacy and safety of *PV*’s active constituents in humans.

**Figure 8:**
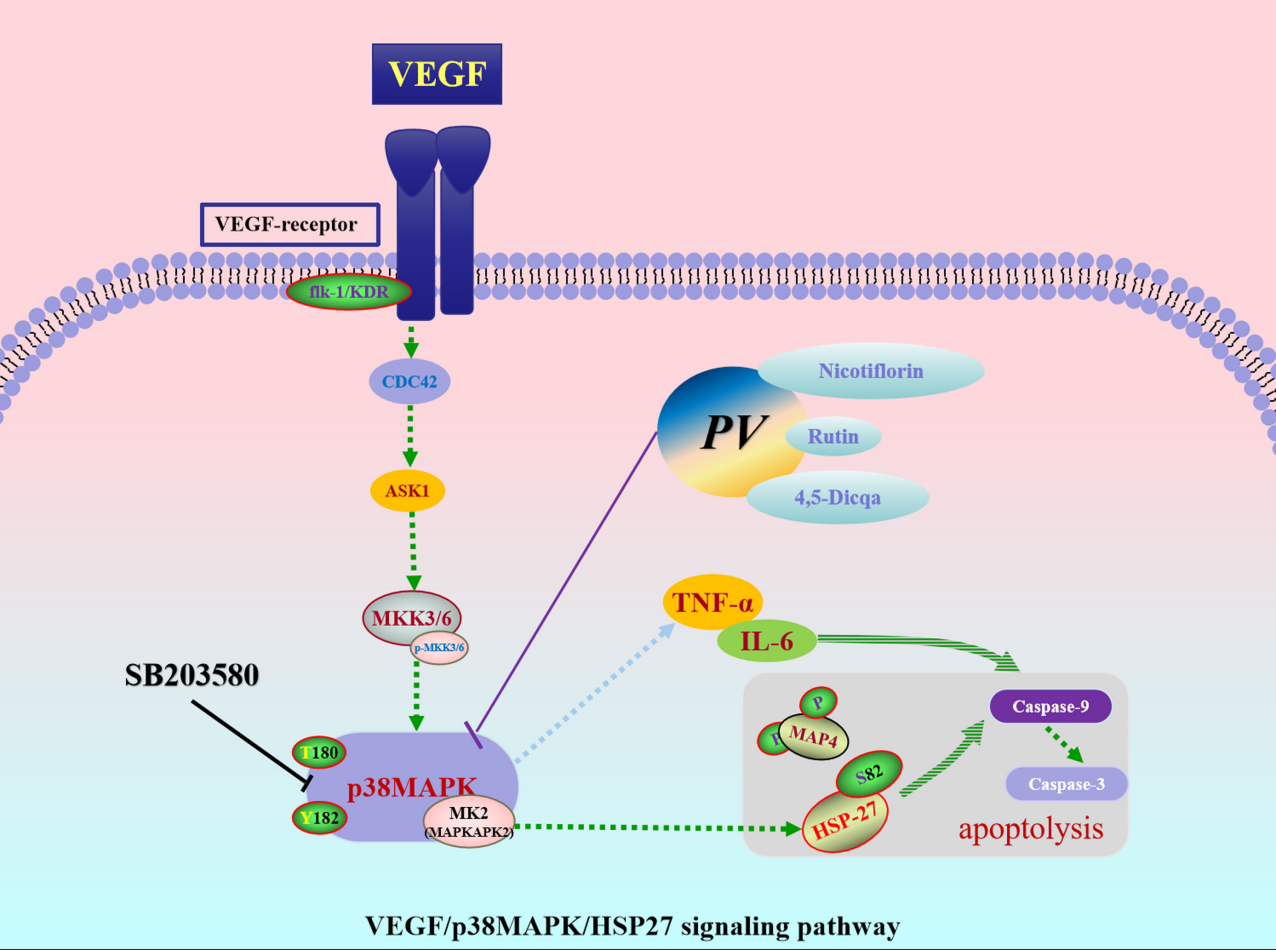
The underlying mechanism of *PV* against CRC.

To sum up, this study systematically identified and validated the VEGF/p38MAPK/HSP27 signaling axis as a pivotal pathway in tumor promoting inflammation. VEGF, p38MAPK, and HSP27 were confirmed as essential therapeutic targets within this pathway. Rutin, nicotiflorin, and 4,5-Dicqa, screened from *PV*, exhibited strong binding affinities to these targets. These findings offer both theoretical and experimental evidence for the development of novel therapeutics targeting tumor promoting inflammation (**Figure 8**).

## 5 Conclusions

In summary, this study systematically identified and validated the pro-tumorigenic inflammatory signaling pathway VEGF/p38MAPK/HSP27, highlighting VEGF, p38MAPK, and HSP27 as key regulatory targets. Based on this, three active compounds rutin, nicotiflorin, and 4,5-Dicqa were screened from *PV*, exhibiting high binding affinities to these targets. MD simulations further confirmed the stability of these compound-target interactions and their favorable drug-like properties. Collectively, our findings elucidate the pivotal role of the VEGF/p38MAPK/HSP27 axis in inflammation driven colorectal cancer progression and validate rutin, nicotiflorin, and 4,5-Dicqa from *PV* as promising lead compounds. This work provides a theoretical foundation for the development of anti-inflammatory therapeutics targeting tumor promoting inflammation.

## Data Availability

The datasets presented in this study can be found in online repositories. The names of the repository/repositories and accession number(s) can be found in the article/**Supplementary Material**.
