# Supplementary material for: Identification and validation of the VEGF/p38MAPK/HSP27 pro-tumor inflammatory pathway: screening of active components from Patrinia villosa and evaluation of their drug-likeness
Source: Front Immunol. 2025 Aug 14;16:1631031. doi: 10.3389/fimmu.2025.1631031 (PMC12391121; doi:10.3389/fimmu.2025.1631031)
Supplement: Supplementary file 1 [file Table1.docx]

***Supplementary***

**MST Experiment Ligand Starting Concentrations and Dilution Scheme**

**VEGFA-*PV* extract:**

Target protein stock concentration: 1.5 µM, diluted to 400 nM.

Ligand starting concentration: 500 µM.

Ligand was serially diluted 1:2 over 16 steps by transferring 10 µl each step. The last 10 µl in tube 16 was discarded to keep equal volumes.

Then, 10 µl of 400 nM target protein was added to each dilution and mixed before measurement.

**VEGFA-Rutin:**

Target protein stock concentration: 1.5 µM, diluted to 400 nM.

Ligand starting concentration: 500 µM.

Ligand was serially diluted 1:2 over 16 steps by transferring 10 µl each step. The last 10 µl in tube 16 was discarded to keep equal volumes.

Then, 10 µl of 400 nM target protein was added to each dilution and mixed before measurement.

**VEGFA-Nicotiflorin:**

Target protein stock concentration: 1.5 µM, diluted to 400 nM.

Ligand starting concentration: 500 µM.

Ligand was serially diluted 1:2 over 16 steps by transferring 10 µl each step. The last 10 µl in tube 16 was discarded to keep equal volumes.

Then, 10 µl of 400 nM target protein was added to each dilution and mixed before measurement.

**VEGFA-4,5-Dicqa:**

Target protein stock concentration: 1.5 µM, diluted to 400 nM.

Ligand starting concentration: 500 µM.

Ligand was serially diluted 1:2 over 16 steps by transferring 10 µl each step. The last 10 µl in tube 16 was discarded to keep equal volumes.

Then, 10 µl of 400 nM target protein was added to each dilution and mixed before measurement.

**VEGFA-SB203580:**

Target protein stock concentration: 1.5 µM, diluted to 400 nM.

Ligand starting concentration: 500 µM.

Ligand was serially diluted 1:2 over 16 steps by transferring 10 µl each step. The last 10 µl in tube 16 was discarded to keep equal volumes.

Then, 10 µl of 400 nM target protein was added to each dilution and mixed before measurement.

**p38MAPK-*PV* extract:**

Target protein stock concentration: 1.5 µM, diluted to 400 nM.

Ligand starting concentration: 1 mM.

Ligand was serially diluted 1:2 over 16 steps by transferring 10 µl each step. The last 10 µl in tube 16 was discarded to keep equal volumes.

Then, 10 µl of 400 nM target protein was added to each dilution and mixed before measurement.

**p38MAPK-Rutin:**

Target protein stock concentration: 1.5 µM, diluted to 400 nM.

Ligand starting concentration: 500 µM.

Ligand was serially diluted 1:2 over 16 steps by transferring 10 µl each step. The last 10 µl in tube 16 was discarded to keep equal volumes.

Then, 10 µl of 400 nM target protein was added to each dilution and mixed before measurement.

**p38MAPK-Nicotiflorin:**

Target protein stock concentration: 1.5 µM, diluted to 400 nM.

Ligand starting concentration: 10 µM.

Ligand was serially diluted 1:2 over 16 steps by transferring 10 µl each step. The last 10 µl in tube 16 was discarded to keep equal volumes.

Then, 10 µl of 400 nM target protein was added to each dilution and mixed before measurement.

**p38MAPK-4,5-Dicqa:**

Target protein stock concentration: 1.5 µM, diluted to 400 nM.

Ligand starting concentration: 50 µM.

Ligand was serially diluted 1:2 over 16 steps by transferring 10 µl each step. The last 10 µl in tube 16 was discarded to keep equal volumes.

Then, 10 µl of 400 nM target protein was added to each dilution and mixed before measurement.

**p38MAPK-SB203580:**

Target protein stock concentration: 1.5 µM, diluted to 400 nM.

Ligand starting concentration: 500 µM.

Ligand was serially diluted 1:2 over 16 steps by transferring 10 µl each step. The last 10 µl in tube 16 was discarded to keep equal volumes.

Then, 10 µl of 400 nM target protein was added to each dilution and mixed before measurement.

**HSP27-*PV* extract:**

Target protein stock concentration: 1.5 µM, diluted to 400 nM.

Ligand starting concentration: 500 µM.

Ligand was serially diluted 1:2 over 16 steps by transferring 10 µl each step. The last 10 µl in tube 16 was discarded to keep equal volumes.

Then, 10 µl of 400 nM target protein was added to each dilution and mixed before measurement.

**HSP27-Rutin:**

Target protein stock concentration: 1.5 µM, diluted to 600 nM.

Ligand starting concentration: 500 µM.

Ligand was serially diluted 1:2 over 16 steps by transferring 10 µl each step. The last 10 µl in tube 16 was discarded to keep equal volumes.

Then, 10 µl of 600 nM target protein was added to each dilution and mixed before measurement.

**HSP27-Nicotiflorin:**

Target protein stock concentration: 1.5 µM, diluted to 600 nM.

Ligand starting concentration: 300 µM.

Ligand was serially diluted 1:2 over 16 steps by transferring 10 µl each step. The last 10 µl in tube 16 was discarded to keep equal volumes.

Then, 10 µl of 600 nM target protein was added to each dilution and mixed before measurement.

**HSP27-4,5-Dicqa:**

Target protein stock concentration: 1.5 µM, diluted to 600 nM.

Ligand starting concentration: 300 µM.

Ligand was serially diluted 1:2 over 16 steps by transferring 10 µl each step. The last 10 µl in tube 16 was discarded to keep equal volumes.

Then, 10 µl of 600 nM target protein was added to each dilution and mixed before measurement.

**HSP27-SB203580:**

Target protein stock concentration: 1.5 µM, diluted to 600 nM.

Ligand starting concentration: 100 µM.

Ligand was serially diluted 1:2 over 16 steps by transferring 10 µl each step. The last 10 µl in tube 16 was discarded to keep equal volumes.

Then, 10 µl of 600 nM target protein was added to each dilution and mixed before measurement.
